# 265. *Cryptococcal* disease in *Covid-19* infection: Case series from South Texas

**DOI:** 10.1093/ofid/ofac492.343

**Published:** 2022-12-15

**Authors:** Sumbal Babar, Marcos I Restrepo, Delvina Ford, Jose Cadena-Zuluaga

**Affiliations:** University of Texas, San antonio, Texas; South Texas Veterans Health Care System, San Antonio, Texas; South Texas Veterans Health Care System, San Antonio, Texas; South Texas Veterans Health Care System, San Antonio, Texas

## Abstract

**Background:**

Covid-19 infection is associated with a lack of immune resilience that may be magnified using immunomodulators to suppress the cytokine storm, facilitating the emergence of opportunistic infections. We describe five cases of cryptococcal infection complications among Covid-19 hospitalized patients.

**Methods:**

This was a retrospective cohort study based on chart review performed at the Audie Murphy Veteran Affairs Hospital from 8/2020 to 8/2021; a level 1A facility with 232 beds and an active bone marrow transplantation program. We included patients aged ≥ 18 with a diagnosis of Covid-19 and subsequent Cryptococcal infection based on cultures or antigen testing.

**Results:**

Our patients were all male with ages ranging between 64 to 80 years. Three had underlying type II diabetes, hypertension, and two had end-stage renal disease. Only one had underlying immunosuppression with hydroxychloroquine for rheumatoid arthritis and one had underlying cirrhosis. Four patients had disseminated disease/fungemia while one had localized pulmonary disease. All the cases had low CD4 counts (158-300) and low CD8 counts (92-290). Two of the fungemia cases were diagnosed by blood culture and the other two by serum cryptococcus antigen test. All the patients had received corticosteroids with or without remdesivir, while one received additional tocilizumab, one baricitinib and one convalescent plasma infusion. Four cases of fungemia received liposomal amphotericin B and three of them received additional flucytosine. The patient with cryptococcal pulmonary disease received only fluconazole. Four patients expired at 28 days after diagnosis, only one recovered and is still alive at 1-year follow up.

**Table 1.**
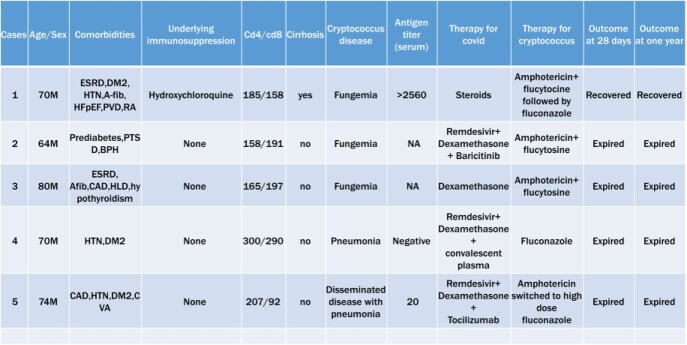
Case details.

ESRD: end stage renal disease; DM-2: diabetes mellitus type ; HTN: hypertension; A-fib: Atrial Fibrillation; HFpEF: heart failure with preserved ejection fraction; PVD: peripheral vascular disease; RA: rheumatoid arthritis; PTSD: post traumatic stress disorder; BPH: benign prostatic hyperplasia; CAD: coronary artery disease; HLD: hyperlipidemia; CVA: cerebrovascular accident.

**Conclusion:**

Cryptococcus infection has been described among patients with Covid-19 during the pandemic. This may be due to immunosuppression caused by the Covid-19 infection and its related-treatments. Most of our patients presented with disseminated cryptococcus infection complicating covid-19 with resulting high mortality rates. Low CD4/CD8 counts and corticosteroid use were documented in all cases. Further studies are needed to better characterize at-risk patients for cryptococcal infection that may benefit from cryptococcal prophylaxis.

**Disclosures:**

**All Authors**: No reported disclosures.

